# Risk for African Swine Fever Introduction Into Kazakhstan

**DOI:** 10.3389/fvets.2021.605910

**Published:** 2021-02-11

**Authors:** Daniella N. Schettino, Sarsenbay K. Abdrakhmanov, Kanatzhan K. Beisembayev, Fedor I. Korennoy, Akhmetzhan A. Sultanov, Yersyn Y. Mukhanbetkaliyev, Ablaikhan S. Kadyrov, Andres M. Perez

**Affiliations:** ^1^Department of Veterinary Population Medicine, Center for Animal Health and Food Safety, College of Veterinary Medicine, University of Minnesota, St. Paul, MN, United States; ^2^Saken Seifullin Kazakh Agrotechnical University, Nur-Sultan, Kazakhstan; ^3^FGBI “Federal Centre for Animal Health” (FGBI “ARRIAH”), Vladimir, Russia; ^4^Kazakh Scientific Research Veterinary Institute, Almaty, Kazakhstan

**Keywords:** risk analysis, African swine fever, Kazakhstan, epidemic, risk map, conjoint analysis

## Abstract

African swine fever (ASF) is a disease of swine that is endemic to some African countries and that has rapidly spread since 2007 through many regions of Asia and Europe, becoming endemic in some areas of those continents. Since there is neither vaccine nor treatment for ASF, prevention is an important action to avoid the economic losses that this disease can impose on a country. Although the Republic of Kazakhstan has remained free from the disease, some of its neighbors have become ASF-infected, raising concerns about the potential introduction of the disease into the country. Here, we have identified clusters of districts in Kazakhstan at highest risk for ASF introduction. Questionnaires were administered, and districts were visited to collect and document, for the first time, at the district level, the distribution of swine operations and population in Kazakhstan. A snowball sampling approach was used to identify ASF experts worldwide, and a conjoint analysis model was used to elicit their opinion in relation to the extent at which relevant epidemiological factors influence the risk for ASF introduction into disease-free regions. The resulting model was validated using data from the Russian Federation and Mongolia. Finally, the validated model was used to rank and categorize Kazakhstani districts in terms of the risk for serving as the point of entry for ASF into the country, and clusters of districts at highest risk of introduction were identified using the normal model of the spatial scan statistic. Results here will help to allocate resources for surveillance and prevention activities aimed at early detecting a hypothetical ASF introduction into Kazakhstan, ultimately helping to protect the sanitary status of the country.

## Introduction

African swine fever (ASF) is a viral disease of pigs, affecting members of the *Suidae* family (domestic pigs or wild boars) without differentiation of age or sex. ASF is caused by a large enveloped, double-stranded deoxyribonucleic acid (DNA) arbovirus that belongs to the *Asfivirus* genus of the *Asfarviridae* family and that is generically referred to as ASF virus (ASFv). ASFv infection has an impact on international trade in pigs and pork products, being a threat to global food security; hence, the disease is notifiable to the World Organization for Animal Health (OIE). ASF epidemics also represent a public health issue because they disrupt the value chain and access to international markets, limiting food access to the population in affected regions and trade partners (1–4). Control measures for ASF are based on biosecurity measures as neither a licensed vaccine nor any treatments are currently available (5).

The ASFv was introduced in 2007 into Georgia, from where the virus spread throughout the Caucasus region (Armenia and Azerbaijan) and the Russian Federation, where the disease became endemic. ASF was subsequently reported in Ukraine and Belarus in July 2012 and June 2013, respectively. In January 2014, ASF reached Eastern Europe, where it spread throughout Estonia, Latvia, Lithuania, Poland, Belgium, Bulgaria, Moldova, Czech Republic, Hungary, Romania, Slovakia, Serbia, Greece, and Germany, affecting wild boars and, in some countries, domestic pigs (3–10). In addition, since 2017, ASF has rapidly spread eastward, with the Russian Federation registering new cases in Eastern Siberia followed by China in 2018; in 2019 Mongolia, Vietnam, Cambodia, Democratic People's Republic of Korea (North Korea), Republic of Korea (South Korea), Lao People's Democratic Republic, Myanmar, Philippines, Timor-Leste, and Indonesia, and in 2020, India and Papua New Guinea also registered cases of ASF. Since 2018, more than 7,300,000 pigs were culled or destroyed in Asia, causing far-reaching economic losses to the region. The unprecedented ASFv spread through Asia and Europe has resulted in great concern for many free countries and regions worldwide (1, 11).

Kazakhstan is a land-locked country located in the transition of Eastern Europe and Central Asia, sharing extensive borders with three countries (Russian Federation, Mongolia, and China) that have been infected by the ASFv. The Kazakh domestic pig sector is relatively small, with ~936,300 pigs and an average density of 0.34 pigs/km^2^ (12). Nevertheless, there is still a potential for increasing the exporting of pork products in association with bans imposed to ASF-infected countries and the consequent increase in demand in importing markets. For those reasons and given that Kazakhstan is still free from the disease, there is an urgent need to increase preparedness for enhancing the chances of early detecting and mitigating a hypothetical ASFv introduction into the country. Because ASF has never been reported in Kazakhstan, there is no information on the socioeconomic or environmental factors associated with the disease spread in the country. For that reason, the allocation of resources in preventive measures that are effective in minimizing the risk of disease incursion is particularly challenging in Kazakhstan.

ASF may be introduced into free areas through different pathways, such as trade of live pigs and pork products, wild boar transboundary movements, and contacts with free-ranging pigs, fomites, and vehicles. The objective of this paper was to identify the areas of Kazakhstan that are most likely to serve as port of entry for a hypothetical ASFv incursion into the country. Results will help the public veterinary authority of Kazakhstan to selectively allocate financial and human resources to target surveillance activities in districts with the highest predicted risk for disease introduction. Additionally, the methodological approach applied here may be used for ranking regions in ASF-free countries located in affected regions worldwide, with the ultimate goal of designing and implementing surveillance programs to prevent and mitigate the impact of the disease (13, 14).

## Materials and Methods

### Data Sources

Because data on the distribution of the susceptible swine population at the district level in Kazakhstan were not available, a country-wise survey was undertaken, aimed at the creation of a national database of pig-related operations. The survey was conducted in 2018–2019 as a series of trips in close collaboration with regional authorities and veterinary services. Locations of all facilities related to the swine industry were georeferenced, and relevant attributes were recorded. The work resulted in the construction of a unique national database of pig holdings as well as slaughterhouses, meat storage, and processing facilities and retail stores. Additionally, data on other relevant variables, as number of pigs per farm and type of pig production based on the ownership of the farms, were compiled and organized in *ad hoc* databases. The database enabled the calculation of pig density and backyard farming share for Kazakhstan used in the present study. Additionally, the estimated wild boar density of Kazakhstan was retrieved from the “Forestry and Wildlife Committee Ministry of Ecology, Geology and Natural Resources of the Republic of Kazakhstan” website (15). The sources of other data used here are provided later at **Table 2**.

### Analytical Approach

Conjoint analysis, which is a marketing research tool used in surveys aimed at capturing the best fit decision of costumers and determining tradeoffs (16), was used in the current study. Districts in a hypothetical ASF-free country located in an ASF-infected region were designed using a factorial design to balance the distribution of epidemiological features hypothesized to influence the risk for ASF introduction. Subsequently, ASF experts were asked to rank those hypothetical districts in terms of the likelihood of serving as port of entry for the disease into the country. An ordinal logistic regression model was run to estimate the relative weight that the experts implicitly gave to each of the variables, as approximated by the value of the regression coefficients. The regression coefficients were then validated using data from the Russian Federation and Mongolia. Finally, the model was used to project the risk in Kazakhstani districts, and high-risk clusters were identified using the spatial scan statistics, to help inform the regionalization of surveillance activities in the country.

#### Conjoint Analysis–Questionnaire and Selection of Variables

A hypothetical ASF-free country was divided into 10 districts using a combination of epidemiological factors hypothesized to influence the risk for ASFv introduction. The 10 districts were designed so that eight of them were created using a factorial design to balance the distribution of epidemiological factors, and two of them represented the scenarios of best and worst possible combination of factors, in terms of their expected risk for the disease (Table 1). A factorial design considers input variables as a factor, where they are combined, and different “treatments” are generated, allowing comparison of the effect of these factors in the independent variable (here, the introduction of ASF) (17). The selection of factors hypothesized to influence the risk was based on previous experience of the authors and supported by a literature search. Pig density, estimated wild boar density, and backyard farming were chosen with the objective of capturing the influence associated with the distribution of the susceptible population. During the ASF outbreaks in Russian Federation, for example, pig population density was identified as an important risk factor for the disease (14, 18). Wild boars can also be responsible for transboundary ASF spread due to their natural dispersal ecology in search of new territory (7, 13, 19). Swill feeding is considered a relatively common practice in many backyard farming systems, which, in addition to limited biosecurity in those types of farms, has been associated with a high risk for the disease (4, 20). Shared border (yes/no) and border length with an infected territory were included because of the risk for movement of infected animals, illegal trade or movement of infected pork, and infected vehicles and other fomites. Finally, human density and road density were included as a proxy for the movement of people, given that travelers can carry contaminated or infected goods and because ASFv can survive for extended periods of time in the environment and in pork products (18, 21) (Table 2).

**Table 1:** A hypothetical African swine fever (ASF)-free country was divided into 10 districts that were characterized in terms of the risk for an ASF introduction using a list of epidemiological factors hypothesized to influence the risk and a factorial design.

**Region**	**Pig density**	**Estimated wild boar density**	**Backyard farming share**	**Share border with ASF-infected country**	**Border length**	**Road density**	**Human population density**	RANK (1–10)^*^
A	Low	Low	Low	No	N/A	High	High	
B	Low	High	High	Yes	Long	High	Low	
C	Low	Low	Low	No	N/A	Low	Low	
D	High	High	Low	No	N/A	High	Low	
E	Low	Low	Low	Yes	Short	Low	Low	
F	High	Low	High	No	N/A	Low	Low	
G	High	High	High	Yes	Long	High	High	
H	Low	High	High	No	N/A	Low	High	
I	High	High	Low	Yes	Long	Low	High	
J	High	Low	High	Yes	Short	High	High	

*The values used to categorize the variables are described in Table 2*.

* *Where 1 means the Lowest Risk and 10 the Highest Risk*.

**Table 2:** Epidemiological factors hypothesized to influence the risk for African swine fever (ASF) were categorized as dichotomous variables considering the values observed in selected countries and regions.

**Risk factor**	**Categories and explanations**	**Reference values**	**Data source**
Pig density (heads/km^2^)	≤2—LOW >2—HIGH	a. Mongolia: from 0.02 to 0.1 with a mean of 0.05 ± 0.03 b. Russian Federation: from 0 to 168.6 with a mean of 9.5 ± 19.4 c. China: from 0 to 363 with a mean of 133 ± 103	Gridded Livestock of the World (GLW 3). Gilbert et al. (22) (https://dataverse.harvard.edu/dataset.xhtml?persistentId=10.7910/DVN/33N0JG)
Estimated wild boar density (heads/km^2^)	≤ 0.03—LOW >0.03—HIGH	a. Mongolia: from 0.01 to 0.05 b. Russian Federation: from 0 to 0.3 with a mean of 0.04 ± 0.05 c. China: from 0 to 2.3 with a mean of 0.2 ± 0.4 d. Most of European countries: from 0.5 to 10	Mongolia, Russian Federation and Europe: Pittiglio et al. (23) China: Hongxuan (24)
Backyard farming share (share of the pig population kept in backyards)	≤10%—LOW >10%—HIGH	a. Russian Federation: 16.5%; b. China: 35% c. Georgia: close to 100%	Russian Federation: Federal State Statistic Service (https://eng.gks.ru/) (25) China: Cheng et al., 2011 (26) Georgia: Beltran-Alcrudo et al. (27)
Border length with an ASF-infected country (km)	≤200 – LOW >200 – HIGH	a. Between Belgium and Germany ~110 km b. Between Ukraine and Poland ~400 km; c. Between Russian Federation and China ~3,000 km;	Data: Esri Data and Maps (28) Computed with ArcGIS
Road density–density of major automobile routes (km^1^)	≤0.1 – LOW >0.1 – HIGH	a. Mongolia: from 0.003 to 0.037 b. Russian Federation: from 0.001 to 0.183 c. China: from 0.05 to 0.31 d. USA: from 0.002 to 0.45 e. Poland: from 0.07 to 0.53	Data: Esri Data and Maps, 2020. (28) Computed with ArcGIS
Human population density (persons/km^2^)	≤10 – LOW >10 – HIGH	a. Mongolia: from 0.28 to 9.3 b. Russian Federation: from 0.37 to 345 c. USA: from 0.4 to 409 d. Poland: from 46 to 806 e. China: 198 to 5,597	Gridded Population of the World (GPW), v4.10 [Center for International Earth Science Information Network—CIESIN (29)]

#### Selection of Experts

The questionnaire listing the hypothetical scenario described above was shared with three OIE Reference Laboratories Centers for ASF (South Africa, Spain, United Kingdom), and the National Reference Laboratory of the Russian Federation in Pokrov, which was selected due to its regional experience on ASF both in wild boars and domestic pigs. The four Reference Centers for ASF were asked to provide names for individuals that would have sufficient knowledge and experience to rank the hypothetical districts in terms for their risk for an ASFv introduction. Snowball sampling (30) was used to designate experts, defined as those individuals that were mentioned at least by two reference centers. A list of 12 experts was identified and was invited to rank the 10 hypothetical districts in terms of the risk for an ASFv incursion, so that #1 and #10 denoted the districts with the lowest and highest risk of becoming ASF-infected, respectively. A table with some definitions and reference values was provided to the experts for helping them understand the values that were used for categorizing the variables (Table 2). Most (*n* = 11, 92%) experts accepted the invitation and answered the questionnaire, which was de-identified prior to data introduction into a master database for analysis.

#### Predictive Model

An ordinal logistic regression (OLR), proportional odds model was fitted to the answers provided by the experts so that

$$\begin{array}{l} \text{ln}=\frac{p\left(Y\geq j\right)}{p\left(Y<j\right)}=\beta_{0}^{\left(j\right)}+\beta_{J}X,\text{ where} \end{array}$$

Y was the dependent variable “score,” as provided by the experts, and so that the score had J categories with j designating categories from 1 to J (i.e., *j* = 1, …, 10). β_0_ was the intercept, and β_*j*_ denoted the coefficients for the independent variables X, which were the epidemiological factors used to characterize each of the hypothetical districts (31). Variables were screened for collinearity prior to their introduction as candidate predictors in the model, and the final model was selected using Akaike's information criterion (AIC).

#### Model Validation and Predictions for Kazakhstan

ASF-infected countries in Central Asia and Eastern Europe (*n* = 14 at the time when this manuscript was written in December 2020, Table 3) were considered as initial candidates for the validation of the model because countries in those regions are culturally, socially, and politically, relatively similar to Kazakhstan, compared to countries in other regions (32–34). Because Kazakhstan is a large country (9th largest in the world) and because the size of the units at which data are aggregated may influence results, the five largest countries (Poland, Germany, Ukraine, Mongolia, and the Russian Federation) from the initial pool of fourteen were subsequently selected as candidate countries for validation. Values for the variables used as risk factors in the model were collected for the five countries at the subnational level and compared with those observed in Kazakhstan (23, 35–37) (Table 4). Poland, Germany, and Ukraine were eliminated as candidate countries for the validation because they are substantially smaller (ranking #69, #63, and #45 in globally size countries, respectively) and also because of the differences in the distribution of values for all assessed variables compared to Kazakhstan–i.e., in general, districts in Ukraine, Germany, and Poland have a higher share of backyard farming and have a higher density of human, domestic pigs, and estimated wild boar density than Kazakhstan. Subsequently, only Mongolia and the Russian Federation were considered adequate for the validation, even acknowledging the differences that exist between those countries and Kazakhstan.

**Table 3:** Comparison of number of oblasts/districts, and country extension (area and world rank) between Kazakhstan and ASF infected countries in Eastern Europe and central Asia.

**Country**	**Number of admin 2 units (Oblasts/Districts)**	**Area (sq km)**	**Area (World rank)**
Bulgaria	28	110,993	103
Estonia	15	45,227	129
Germany	38	357,114	63
Hungary	20	93,036	108
Latvia	119	64 589	122
Lithuania	10	65,301	121
Moldova	47	33,846	135
Poland	16	312,696	69
Romania	42	238,391	80
Russia	82	17,098,246	1
Serbia	25	88,361	111
Slovakia	8	49,034	127
Ukraine	27	603 549	44
Mongolia	22	1,564,110	18
Kazakhstan	173	2,724,900	9

*Source: United Nations Statistics Division (32)*.

**Table 4:** Distributions of district and regions/oblasts for the countries considered as candidate countries for model validation for predict the risk of introduction of ASF in Kazakhstan.

**Variables (Risk factors)**	**Value**	**Kazakhstan**	**Ukraine**	**Russia**	**Mongolia**	**Germany**	**Poland**
Backyard farming share	High	58	25	55	20	22	16
	Low	115	2	27	2	16	0
Human pop. Density	High	41	27	56	3	38	16
	Low	132	0	26	19	0	0
Estimated wild boar density	High	34	25	41	9	36	16
	Low	139	2	41	13	2	0
Domestic pig density	High	12	25	43	1	34	16
	Low	161	2	39	22	4	0
Road density	High	40	3	4	0	38	12
	Low	133	24	78	22	0	4
Share border with ASF infected country	Yes	52	8	7	8	5	3
	No	121	19	75	14	33	13

For the validation, the regression coefficients obtained from the OLR model were used as weighting factors for the data collected in both Mongolia and the Russian Federation to identify the three districts (regions or oblasts) predicted to be at the highest risk for introduction of ASFv when those countries were free from the disease (Table 5). The results, which indicated the districts that would have been identified by our model and the expert opinion elicited here at the highest risk for ASFv introduction into the Russian Federation and Mongolia, were compared to the districts through which the disease was introduced into those countries when they first-became ASF-infected, as recorded by OIE's World Animal Health database (WAHID) (38, 39).

**Table 5:** Association between selected epidemiological factors and risk for introduction of African swine fever (ASF) into a free country located in an infected region, as suggested by elicitation of expert opinion through a conjoint analysis model.

	**Coefficient**	**CI (95%)**	**Odds ratio**	**Std. error**	***p*-value**
Pig density (high)	3.39	2.27, 4.52	33.3	0.58	<0.01
Estimated wild boar density (high)	3.4	2.28, 4.52	33.3	0.57	<0.01
Backyard farming (high)	4.16	3.00, 5.33	50	0.59	<0.01
Share border (yes)	2.34	1.49, 3.19	10.4	0.43	<0.01
Road density (high)	0.67	0.08, 1.44	2	0.39	0.083
Human density (high)	0.55	0.2, 1.3	1.8	0.38	0.148

**Figure 1:**
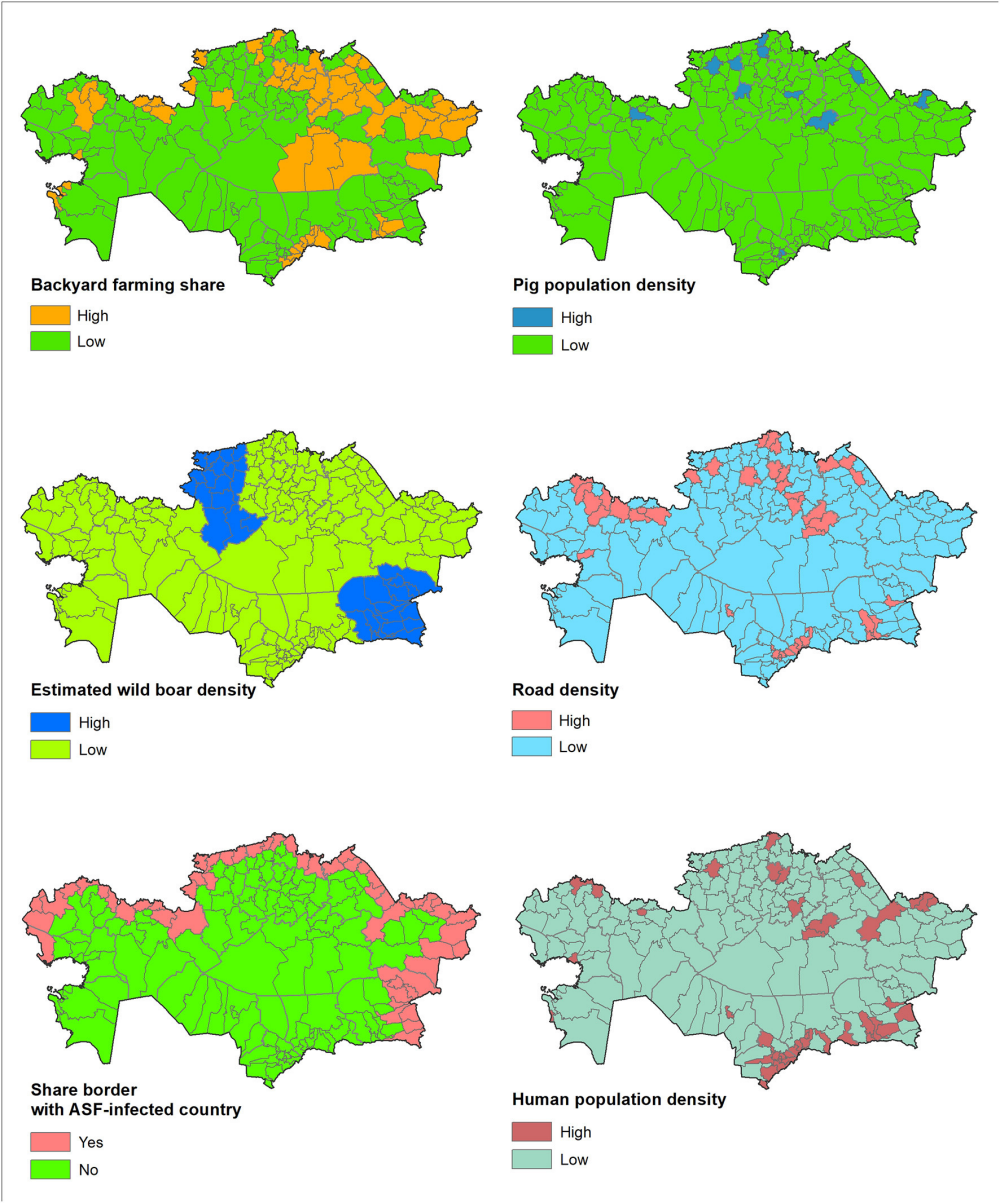
Kazakhstan district-level data for the variables used in the model (backyard farming share, domestic pig density, estimated wild boar density, share-border with ASF-infected country, human population density, and road density).

Finally, the OLR model was applied to the district-level data (pig density, estimated wild boar density, backyard farming, shared border (yes/no), human density, and road density) collected in Kazakhstan to predict the districts at highest risk for disease introduction. Figure 1 depicts the categorization of these district-level data in Kazakhstan. Results were allocated to each of the district centroids, and the normal model of the spatial scan statistic was run to identify clusters of districts in which the predicted risk of introduction of ASFv was significantly (*P* < 0.05) higher than expected under the null hypothesis of even distribution of risk. The normal model of the spatial scan statistic has been described elsewhere (40). Briefly, circles of variable radius are alternatively imposed over the centroids and candidate clusters, including groups of neighboring districts, are identified. The average risk for ASF introduction was computed for each candidate cluster and compared with the expected under the null hypothesis that all observations come from the same distribution. Significance of the deviation of the observed risk, compared to the expected, was estimated for each candidate cluster using Monte Carlo simulation. Results for Kazakhstan were plotted in choropleth maps.

### Modeling Environment

The SPSS software (41) was used for the factorial design of the 10 hypothetical districts. The RStudio Team (2019) version 3.5.3 (42) was used for performing the OLR model, using the packages MASS, tidyverse, and ggbeeswarm. The SaTScan v.9.6 software was used to identify clusters of predicted risk in Kazakhstan (43). ArcGIS 10.7.1 was used for spatial data processing and mapping data and results (44).

## Results

**Figure 2:**
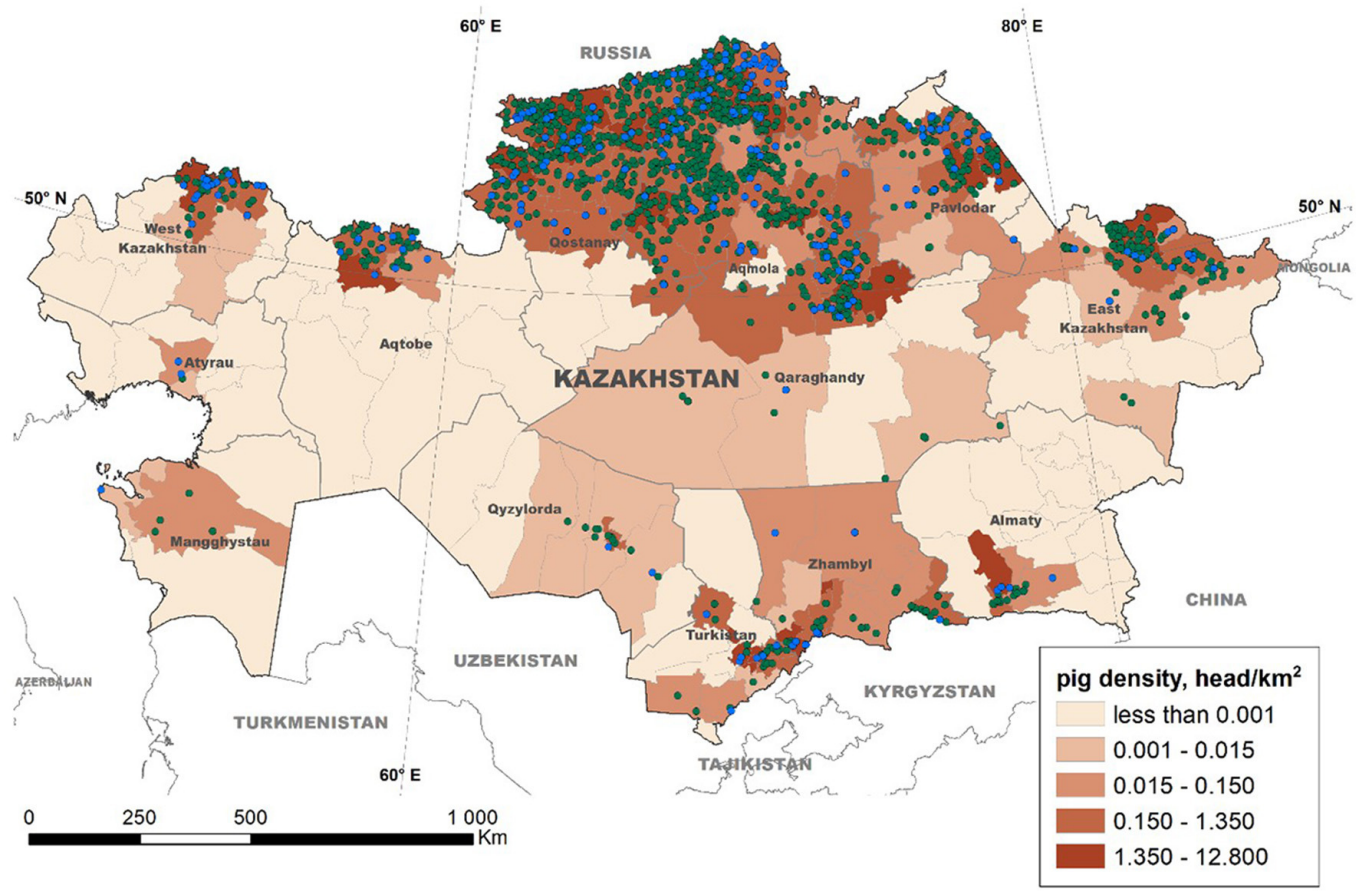
Distribution of swine farms in Kazakhstan. The location of swine operations is indicated and categorized as single-owner farms (green dots) and commercial association-owned (blue dots) farms. The color gradient denotes the pig density (head/sq km) estimated at the district level.

The data collection process led to the registration of 2,021 pig farms throughout Kazakhstan. Based on the legal property form of the farms, most operations (*n* = 1,612, 79.5%) were considered privately owned (i.e., belonging to a single stakeholder) farms, with swine population sizes ranging between 1 and 6,110 pigs (median of 107 pigs). The remaining operations (*n* = 409, 20.5%) were classified as farms belonging to commercial associations, with 1 to 50,775 pigs (median of 47 pigs) (Figure 2). This categorization only reflects the legal property type, as no biosecurity-based classification is currently effective in Kazakhstan. For the purposes of data analysis, we only used pig population per farm to categorize all holdings in “small,” conventionally treated as backyards (<100 pigs), and “large” (more than 100 pigs), consistently with the Food and Agriculture Organization of the United Nations (FAO) definition of backyard production systems as those in which pigs are confined in very simple pens are dependent for their keeper for feed, and the herd is usually small (1–100 animals raised per year) (45).

Because there was a high level of collinearity between border length (km) and shared border (yes/no) with an infected country, the former variable was considered redundant and removed from the model. The factor that experts considered most important in driving the risk for introduction of ASFv into a free district was a high density of backyard farming, followed by high density of pigs and high estimated density of wild boars (Table 5).

Despite that road and human densities were not significantly associated with the score provided by the experts, inclusion of those variables in the final model resulted in the lowest AIC value recorded for any combination of variables (AIC: 343.4), and for that reason, all variables listed in Table 5 were retained in the final model.

**Figure 3:**
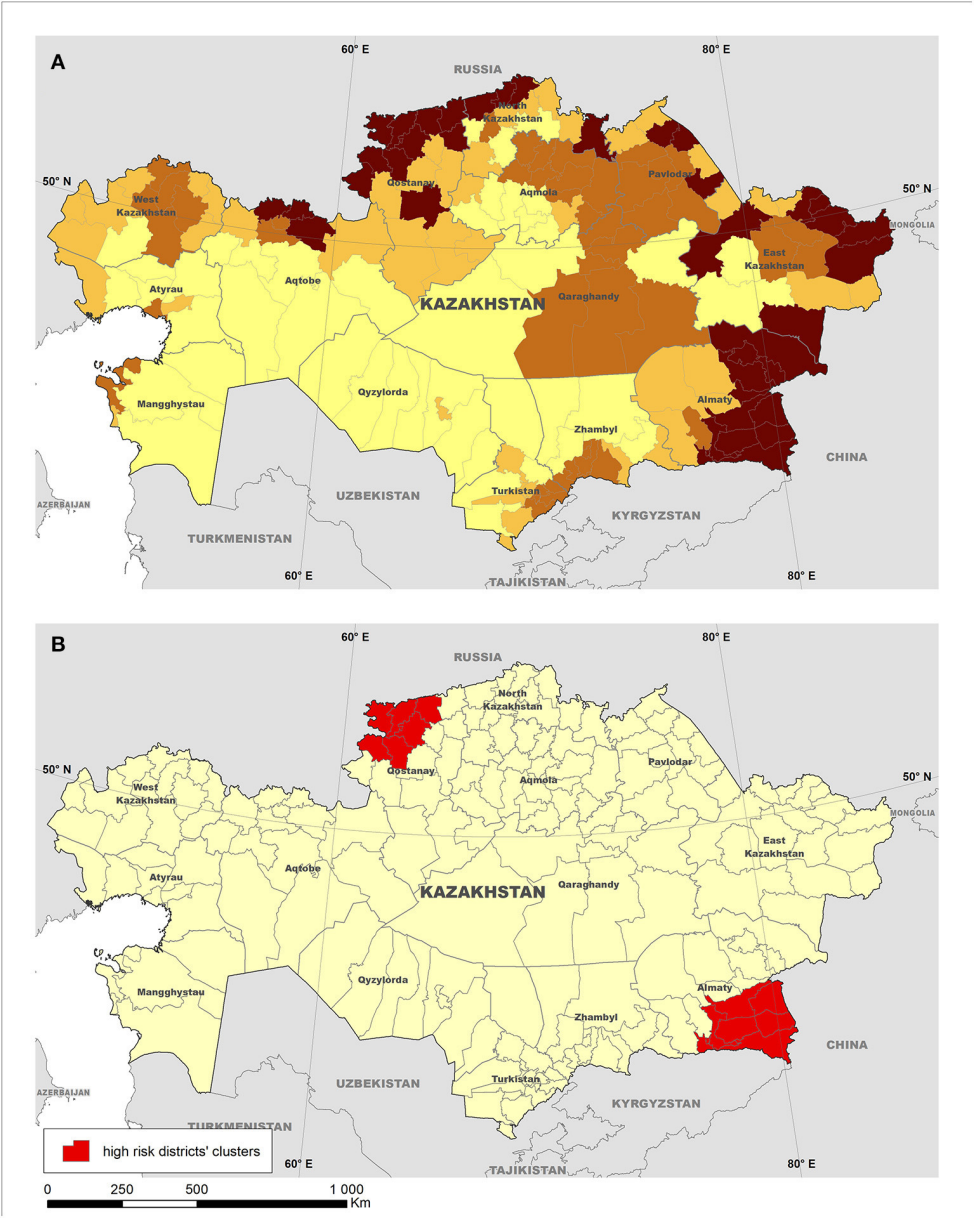
Risk for introduction of African swine fever (ASF) into Kazakhstan estimated using a conjoint analysis model. The map on the top **(A)** depicts districts grouped into four quantiles based on the predicted risk (the darker the shade, the higher the risk), whereas the map on the bottom **(B)** illustrates the location of clusters of high risk for the introduction of ASF into the country detected using the normal model of the spatial scan statistic.

The three Russian Federation districts predicted to be at highest risk for introduction of the disease were the Republic of North Ossetia-Alania, Bryansk Oblast, and the Orenburg Oblast, respectively. Although ASFv was first reported in Chechen Republic in November 2007, which would not have been predicted by our model, the second massive incursion of ASFv into Russian Federation was reported in June 2008 in the Republic of North Ossetia-Alania, followed by cases in Orenburg Oblast in July 2008, coincidentally with the model predictions. For Mongolia, the three districts predicted to be at highest risk of the introduction of ASF were Ulaanbaatar, Bulgan, and Selenge. Coincidently, the three districts had the first occurrence of ASF in January 2019. Subsequently, the resulting model was used to predict the risk for ASFv introduction into Kazakhstan, and two clusters of significantly (*P* < 0.05) high risk for introduction of ASFv in that country were detected using the spatial scan statistic. High-risk clusters were located in the Almaty (southern Kazakhstan) and Kostanay (northern Kazakhstan) regions and include seven and nine districts, respectively (Figure 3).

## Discussion

Following the fall of the Soviet Union and given that the majority of the population of Kazakhstan is Muslim, the number of swine operations in the country has substantially decreased. However, the swine industry of Kazakhstan still supplies the demand for >25% of the population of the country that is not Muslim. Furthermore, the geographical proximity of Kazakhstan with China has increased the country's interest in promoting the production of pork to supply the emerging demand in China associated with the ASF epidemic. The Kazakh Ministry of Agriculture has signed a memorandum of understanding “on inspection, quarantine and veterinary-sanitary requirements for pork exported from Kazakhstan to China” with the Chinese State Technical University, which was considered a first step to promote pork exports into China (46). In order to protect the status of the Kazakh swine industry, it is critical to understand the distribution of the susceptible population and characterize the risks associated with disease status. For the first time, we have conducted here a comprehensive survey of the distribution of swine farms in Kazakhstan, showing its selective concentration in the northern and southern regions of the country (Figure 2).

The relative isolation of Kazakhstan, along with the small size of its pig industry, may have helped the country to avoid the introduction of ASFv, despite the unprecedented spread of the disease through Europe and Asia. However, given that a number of neighboring countries have become ASF-infected, there is a need for supporting Kazakhstan preparedness through the identification of areas at highest risk for ASFv introduction. The results here may help to target surveillance activities to those districts identified at highest risk for disease introduction to increase the sensitivity of the national surveillance system and support the early detection of a hypothetical ASF introduction into the country (Figure 3). The characterization of districts within those clusters as at highest risk for ASFv was driven by the presence of a number of factors that have influenced disease introduction into free regions. Those factors include the size of their domestic pig population and the estimated wild boar population, and their close proximity to ASF-infected countries, which are important to inform the design of targeted surveillance efforts (47). Most importantly, many risk prediction studies suggest wild boars as the highest risk factor involved in ASF transmission (7, 19, 48) for countries in Europe, the Caucasus region, and Central Asia.

Because Kazakhstan has never been infected by the ASFv, there is no historical information that could help the country to categorize districts in terms of their risk for the disease. In the absence of such information, we gathered expert opinion on the factors that have driven the introduction of the disease in free countries of Europe and Asia. The highest risk oblast in the Russian Federation, the Republic of North Ossetia, was the second district infected in the country. Noteworthily, the failure of our model to identify Chechen Republic (the district through which the disease was first introduced into the Russian Federation) was likely due to the social disruption associated with the constitutional war suffered by the region at the time of the epidemic. Such social disruption may have resulted in an unexpected frequent movement of people and contaminated products or food that could not have been predicted by the formulation of our model. Noteworthily, cases in the Chechen Republic were very limited. In contrast, the next affected region, the Republic of North Ossetia, which was predicted at the highest risk for introduction by our model, suffered a large number of cases in domestic pigs and actually may be considered a starting point of the consequent spread of ASF in the Russian Federation. The Republic of North Ossetia is also closely connected with the neighboring regions of Georgia, and ASF transmission was certainly expected here. Similarly, the first introductions of ASF into Mongolia took place at one of the districts identified at highest risk by our model. For those reasons, and in the absence of a prior history of ASF in Kazakhstan, the results of the validation process suggest that the model may help to accurately predict the expected risk for ASFv introduction into the country.

The study here did not assess the likelihood of disease introduction into Kazakhstan. Instead, we ranked the districts through which the disease was most likely to be introduced into the country, given that an incursion effectively occurs. This information is important to inform the design of targeted surveillance efforts in the country. One limitation is that epidemics are typically low probability events, and the realization of those processes is susceptible of being affected by random events, such as the social disruption in Chechenia. For that reason, the risk predicted here would be accurate only if the modeled conditions, reflected by the epidemiological factors weighted by the experts, remain constant in the future. Any variation in those conditions, or if those assumptions would not hold truth for Kazakhstan, may result in a variation of the predicted risk for the country.

In conclusion, the study here provided updated information on the spatial distribution of swine operations in Kazakhstan, along with the prediction of areas at highest risk for introduction of ASFv into the country. Results have been shared with the government of Kazakhstan to support the development of recommendations on prevention and control measures for ASF in the country. This document will define a national strategy to prevent the introduction of ASFv from neighboring countries, and it is intended to become mandatory for implementation at all pig farms in Kazakhstan under the supervision of the national veterinary authority. For those reasons, ultimately, the results will help to sustain the ASFv-free status of Kazakhstan and support the country's vision and efforts to supply international markets.

## Data Availability Statement

The raw data supporting the conclusions of this article will be made available by the authors, without undue reservation, except the pig data from Kazakhstan, which is considered confidential and may be available upon request.

## Author Contributions

DS and SA led study design and analysis, they also did sample collection processes and wrote much of the paper. FK collaborated with the data analysis process and wrote some of the paper. KB, AS, YM, and AK contributed with data collection and organization and wrote some of the paper. AP supervised the design of the study and paper and the data analysis process. All authors contributed to the article and approved the submitted version.

## Conflict of Interest

The authors declare that the research was conducted in the absence of any commercial or financial relationships that could be construed as a potential conflict of interest.
